# Isolation and Functional Characterization of Endophytic Bacteria from Muscadine Grape Berries: A Microbial Treasure Trove

**DOI:** 10.3390/cells14050369

**Published:** 2025-03-03

**Authors:** Meenakshi Agarwal, Mehboob B. Sheikh

**Affiliations:** Center for Viticulture & Small Fruit Research, Florida A&M University, Tallahassee, FL 32317, USA

**Keywords:** muscadine grape, *Vitis rotundifolia*, endophytes, bacteria, biochemical characterization

## Abstract

Muscadine grapes are renowned for their unique traits, natural disease resistance, and rich bioactive compounds. Despite extensive research on their phytochemical properties, microbial communities, particularly endophytic bacteria, remain largely unexplored. These bacteria play crucial roles in plant health, stress tolerance, and ecological interactions. This study represents the first comprehensive effort to isolate, identify, and functionally characterize the bacterial endophytes inhabiting muscadine grape berries using a culture-dependent approach. We isolated diverse bacterial species spanning six genera—*Bacillus*, *Staphylococcus*, *Paenibacillus*, *Calidifontibacillus*, *Curtobacterium*, and *Tatumella*. Microscopic and physiological analysis revealed variations in bacterial morphology, with isolates demonstrating adaptability to varied temperatures. Cluster-based analysis indicated functional specialization among the isolates, with species from *Pseudomonadota* and *Actinomycetota* exhibiting superior plant growth-promoting abilities, whereas *Bacillota* species displayed potential biocontrol and probiotic properties. Among them, *Tatumella ptyseos* demonstrated exceptional plant growth-promoting traits, including indole-3-acetic acid production, nitrogen fixation, phosphate solubilization, and carbohydrate fermentation. Additionally, *Bacillus* spp. showed presumptive biocontrol potential, while *Paenibacillus cineris* emerged as a potential probiotic candidate. The identification of *Calidifontibacillus erzurumensis* as a novel endophytic species further expands the known biodiversity of grape-associated microbes. These findings provide insights into the metabolic diversity and functional roles of muscadine grape-associated endophytes, highlighting their potential for agricultural and biotechnological applications.

## 1. Introduction

The muscadine grape (*Vitis rotundifolia*), native to the southeastern United States, is known for its distinctive flavor profile and suitability for winemaking. Unlike the commonly cultivated bunch grapes (*Vitis vinifera*), muscadine grapes possess unique characteristics, including larger berries with thick skins and large seeds [1,2]. Beyond its sensory appeal, the muscadine grape is notable for its rich phytochemical composition. Previous studies have highlighted the presence of a wide array of bioactive compounds, such as polyphenols and flavonoids, in these grapes [3,4]. These bioactive compounds are known for their potent nutraceutical benefits, including antioxidant, cardiovascular, anti-inflammatory, anti-obesity, anticancer, neuroprotective, and antimicrobial activities [5,6,7,8,9,10]. This rich array of bioactive components has positioned the muscadine grape as a fruit of significant interest not only in the food industry but also in the field of health and wellness.

In addition, muscadine grapes are known for their resilience to diseases caused by fungi and bacteria such as downy mildew, powdery mildew, Anthracnose, and Pierce’s disease with variations among different cultivars compared to bunch grapes [11,12,13,14,15,16]. This resilience is a considerable advantage, especially in regions like where warm and humid climate can promote the growth of organisms that cause fruit rot. Additionally, certain insect pests are also known to cause less damage to muscadine grapes than to other grape varieties [17,18].

Muscadine grapes, like other grapevines, harbor a varied assemblage of endophytic microbes [19]. Endophytes often provide beneficial effects to their host plants, such as fixing nitrogen, solubilizing phosphate, producing phytohormones, improving water and nutrient absorption, enhancing enzymatic reactions, and many more [20,21]. Furthermore, certain endophytic bacteria have also been reported to increase the production and accumulation of significant secondary metabolites in the host [22,23,24,25,26,27,28]. Moreover, endophytes have been known to combat both abiotic and biotic stresses. Certain strains of *Burkholderia phytofirmans* enhance grapevine growth and resistance to cold stress [29] and chilling stress by accumulating trehalose-6-phosphate and trehalose [30]. Additionally, the *Xylella fastidiosa* strain EB92-1, obtained from elderberries, has demonstrated effectiveness as a biocontrol agent against Pierce’s disease in Florida and other commercial vineyards [31].

In addition to their advantages to host plants, endophytes are recognized for their wide application in both animal and environmental contexts. A previous study investigating the endophytic microbial community of *Vitis amurensis* and *Vitis coignetiae* grapes showed the presence of microbes which are often associated with the human microbiome. These include bacterial genera such as *Lactobacillus*, *Enterococcus*, *Bacteroides*, *Streptococcus*, *Escherichia-Shigella*, and *Bacillus*. These species may function as probiotics and influence the prevention of degenerative diseases such as obesity, diabetes, cancer, cardiovascular disorders, malignancies, liver disease, and inflammatory bowel disease [32]. Furthermore, an article highlighted the potential of endophytic bacteria in bioremediation [33]. *Bacillus* sp. could decrease the cadmium by 94% in the presence of industrial metabolic inhibitors such as N′-dicyclohexylcarbodiimide [34]. A role of *Pseudomonas* sp. has been shown in xenobiotic chemical degradation [35].

Collectively, these studies confer that endophytes have the ability to augment host plant’s capacity to withstand different forms of stress. It has been hypothesized that plants may encounter challenges in their survival when they are devoid of endophytic microorganisms. Given the unique phytochemical composition and the ability of muscadine grapes to resist pests and diseases, it is likely that these vines host a distinct community of microbes that contributes to their resilience and traits. Therefore, it is crucial to isolate and identify these microorganisms to understand their mechanisms and potential uses in agriculture, industry, and medicine. To date, there has been no investigation into the isolation of bacterial endophytes from muscadine grapes. A previous study has explored the microbial diversity of muscadine grape skin using non-culture-based method, thus limiting the practical use of microbes [19]. In this study, we aim to isolate, identify, analyze the diversity of, and functionally characterize the bacterial endophytic community that colonizes inside the muscadine berry and can be cultured under laboratory conditions. This study has the potential to promote the natural production of endophytic agents that can enhance grapevine stress resistance and improve the quality of grape-derived products, as well as potentially benefiting other crops and their usage by humans.

## 2. Material and Methods

### 2.1. Grape Berry Sample Collection

Healthy ripe berries from twelve distinct muscadine grape cultivars, ranging in color from bronze to red/purple, were harvested from the vineyard at the Center for Viticulture and Small Fruit Research, Florida Agricultural and Mechanical University, Tallahassee, FL, USA, during the September 2021 vintage. Immediately after harvesting, the berries were transported to the laboratory for processing. Juice was collected from fresh berries and used for brix and pH measurements. Brix was measured using digital refractometer and pH was measured using a pH meter. The experiment was performed in triplicate.

### 2.2. Bacterial Isolation and Purification

For each cultivar, five to six berries were selected and subjected to surface sterilization. In brief, berries were rinsed with sterile water and immersed in 70% ethanol for 1 min, followed by 2.5% sodium hypochlorite for 5 min, and finally rinsed with sterile distilled water. The berries’ skin was removed aseptically and the resulting berries, along with the pulp and seeds, were homogenized to facilitate endophytic bacterial isolation. The prepared homogeneous suspension was inoculated into LB broth (Sigma-Aldrich, St. Louis, MO, USA) and incubated at 30 °C until growth was observed. Following growth, the cultures were serially diluted and plated onto LB agar plates. Colonies with distinct morphologies were selected and streaked onto fresh LB plates to obtain isolated colonies. These isolated colonies were screened through microscopic examination to confirm the presence of pure and single species. Plates and microscopic photographs were taken to record their morphological characteristics like size, shape, color, and colony texture. The confirmed isolates were then preserved according to the method described earlier [36].

### 2.3. Molecular Identification of Isolates

Individual colonies of each isolate were grown separately in the LB broth overnight at 30 °C on a rotary shaker at 200 rpm. The cultures were centrifuged and the resulting pelletized cells were used for the DNA extraction using the ZR fungal/bacterial DNA kit (Zymo Research, Irvine, CA, USA). In total, 20–50 ng of extracted DNA was used for the 16S rDNA amplification using 27 F and 1492 R primers and amplified products were processed for the Sanger sequencing [37] using one forward reaction. The obtained sequences were edited manually and subjected to the Blastn search in the NCBI. The top database hits were used to identify the most probable taxonomic resolution to a species level with at least a 99% confidence interval.

### 2.4. Growth Curve Analysis

The inoculum was prepared by incubating the strains in the LB and MRS broth overnight at 30 °C with shaking at 200 rpm. The following day, the cultures were diluted to an OD_600_ of 0.2. The assay was conducted using LB and MRS broth media, and growth was monitored at temperatures of 25 °C, 30 °C, and 37 °C. OD_600_ measurements were taken every 3 h for up to 48 h [38]. The experiment was performed in triplicate, and the average values along with standard deviations were calculated.

### 2.5. Biochemical Characterization

For all biochemical characterization assays, overnight cultures were grown on MRS agar plates and served as the inoculum source. Phosphate solubilization ability was assessed using Pikovskaya’s (PVK) agar media [39]. Isolates were spot-inoculated onto the agar and incubated at 30 °C for up to 14 days. Phosphate solubilization was evidenced by the formation of a halo around the colonies, and the halo size was quantified by subtracting the colony diameter from the total diameter (colony plus halo). Nitrogen fixation ability was tested by growing the isolates on the nitrogen-free medium called Jensen medium and incubating at 37 °C [40] Strains showing growth on this medium were considered to have ability to fix the atmospheric nitrogen. Indole-3-acetic acid (IAA) production was tested using the method described previously with some modifications [41]. In brief, strains were grown in nutrient broth for 24 h and then inoculated with nutrient broth with 0.5% tryptophan for 3 days at 30 °C with 200 rpm. A portion (1.5 mL) of the culture was transferred to a new test tube and centrifuged at 16 g for 5 min. Then, 1 ml of supernatant was transferred to a new test tube and mixed with an equal volume of Salkowski reagent, vortexed gently, and incubated at 30 °C in the dark for 30 min. Uninoculated medium mixed with Salkowski reagent served as the control. The presence of IAA was detected by measuring pink color development. The color intensity was measured spectrophotometrically at 536 nm.

To test the carbohydrate fermentation, Triple Sugar Iron agar slants, consisting of sucrose, lactose, and glucose and ferrous sulfate, were used. Strains were inoculated by stabbing into the medium and streaking the surface agar slant. Tubes were incubated at 37 °C for 24 h. The slant and butt color were monitored. Red slant and yellow butt were considered to be dextrose fermentation. Yellow slant and yellow butt were indicative of dextrose, lactose, or sucrose. Red slant and red butt were indicative of the absence of carbohydrate fermentation. No change in color was indicative of no fermentation. Oxidase activity was determined by applying colonies to filter paper impregnated with oxidase reagent (Oxidrop liquid oxidase reagent). The development of a blue or purple coloration within 10–20 s was considered to be oxidase-positive. Catalase production was tested using the slide test method, where a small loopful of the culture was mixed with hydrogen peroxide on a glass slide, and the production of effervescence was observed as an indicator of catalase activity.

Motility assay was performed using soft agar tubes [42]. The individual strains were stabbed into the soft agar tube and incubated at 37 °C for 48 h. Strains that exhibited growth radiating outward from the stab line were classified as motile, whereas those with growth confined strictly to the stab line were classified as non-motile. To evaluate DNase activity, isolates were streaked onto DNase agar containing toluidine blue and incubated at 37 °C for 24 h. The presence of a clear zone around the colonies indicated positive DNase activity. Hemolytic activity was assessed by streaking the isolates onto Columbia agar supplemented with 5% (*w*/*v*) sheep blood, followed by incubation at 37 °C for 24 h. Hemolysis was categorized as α-hemolysis (green zones surrounding colonies), β-hemolysis (clear zones), or γ-hemolysis (absence of zones).

### 2.6. Tolerance Assay to Intestinal Fluids, Bile Salts, Simulated Gastric Juice, and Acidic pH

To assess the tolerance of bacterial isolates, active cultures were prepared by growing them in MRS broth at 37 °C overnight. Following incubation, the bacterial cells were harvested by centrifugation at 1500× *g* for 10 min. The resulting cell pellets were then washed once with PBS solution to remove any residual medium, and the cells were subsequently resuspended in fresh PBS solution for further analysis. An aliquot of this suspension was then inoculated at a concentration of 1% (*v*/*v*) into a solution specific to each assay, and incubated at 37 °C for 4 h followed by the cell survival assay.

The intestinal fluid tolerance test was conducted using a simulated intestinal fluid with a pH of 6.8, which was filter-sterilized through a 0.22 µm filter prior to use. For the bile salt tolerance test, MRS broth containing 0.3% (*w*/*v*) bile salt was filtered and used as a medium. To assess tolerance to gastric juice, a simulated gastric fluid of pH 1.0–1.4 containing pepsin at a concentration of 3 mg/mL was employed. For the acid tolerance test, aliquots were resuspended in MRS broth adjusted to pH levels of 4.0, 3.0, and 2.0 using 5 N HCl.

### 2.7. Cell Survival Assay

The viable cell populations were determined using the spread plate method employing 10-fold serial dilutions on MRS agar plates, both before and after incubation/treatment. The percentage survival of the bacteria was calculated by counting the colony-forming units (CFU) using the following formula: % survival = (log CFU of viable cells after incubation/log CFU of initial viable cells inoculated × 100). Each assay was conducted in triplicate, with the mean values calculated and the standard deviation reported. Additionally, a plate MIC method, as described by Agarwal et al. [38], was employed to enhance visibility and enable comparison across all dilutions on a single plate.

### 2.8. Microscopy Imaging

For cell imaging, bacterial strains were grown on LB plates, and a small loop of the culture was suspended in PBS solution. Light and fluorescence microscopy images were taken using the EVOS M5000 (Thermo Fisher Scientific, Carlsbad, CA, USA), as described previously [43]. Bacterial cell membranes were visualized with FM 1-43FX (Thermo Fisher Scientific, Carlsbad, CA, USA), following the protocol detailed by Agarwal et al. [43]. Cell lengths were measured in micrometers using ImageJ 1.54f software, and images were processed with the same software.

### 2.9. Statistical Analysis

All experiments were performed in triplicate, and the data are presented as mean values with error bars indicating the standard deviation. *p* values were calculated using one-way ANOVA, and differences were considered statistically significant at *p* < 0.005. Heatmaps and clustering dendrograms were prepared using R software (R 4.1.0).

## 3. Results

### 3.1. Isolation and Molecular Identification Revealed Presence of Diverse Bacterial Endophytic Species in Muscadine Berries

**Figure 1:**
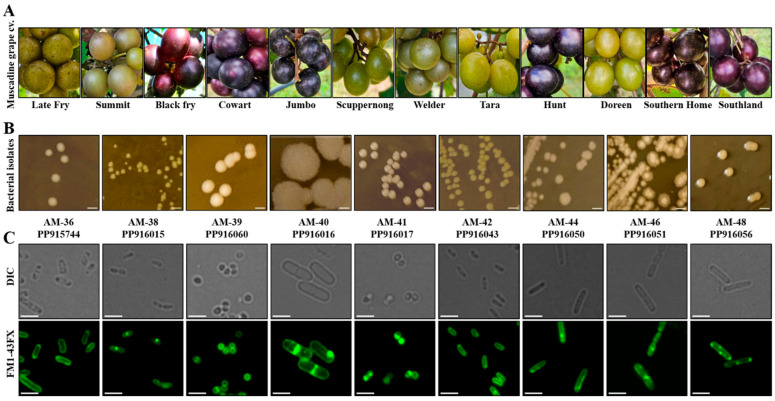
Morphological diversity of bacterial endophytes isolated from different muscadine berry genotypes. (**A**) Representative images of muscadine grape cultivars used in this study. (**B**) Plate images represent colony morphology and color and shape of bacterial endophytes grown on LB agar plates. Scale bar: 2.5 µm. (**C**) Microscopic images of individual bacterial isolates; both DIC and those stained with the membrane marker FM1-43FX shown. Scale bar: 2.5 µm.

A total of twelve different muscadine grape cultivars were chosen, and among these, six cultivars had bronze-colored berries and the remaining six had black-colored berries (Figure 1A). The pH analysis conducted on ripe berries showed a pH range from 3.2 to 3.7, while Brix measurements varied from 13.5 to 17.6 (Table 1). Among the cultivars tested, the cultivar Doreen displayed the lowest Brix value, while the cultivar Jumbo recorded the highest (Table 1). Endophytic bacterial population enrichment in liquid broth and, further, their successive transfer on the agar plate revealed diverse range of bacterial colonies based on appearance. Further molecular identification of differently looking purified individual strains via 16S rRNA coding gene sequencing resulted in the identification of total 15 culturable endophytic bacterial isolates under the tested laboratory condition (Table 1). The identified bacterial types also varied among the cultivars to some extent. Among these, only 2 isolates were Gram-negative, and the remaining 13 strains were Gram-positive (Table 1). Out of 15 isolates, 9 distinct bacterial species were identified, selected for further characterization, and deposited in NCBI, with corresponding accession numbers listed in Table 2.

**Figure 2:**
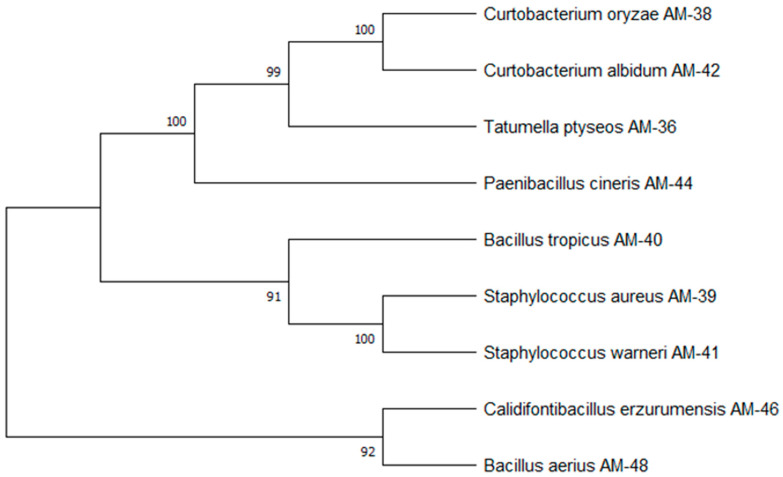
Phylogenetic tree of bacterial endophytes isolated from muscadine berries. The tree illustrates the evolutionary relationships among the bacterial isolates. The tree was constructed using partial 16S rRNA gene sequences, analyzed by the neighbor-joining method. Bootstrap values (expressed as percentages) are shown at branch points, indicating the reliability of the clustering.

The dominant phylum among the isolates was found to be Bacillota, including the strains *Staphylococcus aureus*, *Staphylococcus warneri*, *Bacillus tropicus*, *Paenibacillus cineris*, *Calidifontibacillus erzurumensis*, and *Bacillus aerius*. The remaining three strains belonged to the phyla Actinomycetota (i.e., Curtobacterium *oryzae* and *Curtobacterium citreum*) and Pseudomonadota (*Tatumella ptyseos*) (Table S1). A phylogenetic tree based on partial 16S rDNA sequences was constructed to examine the relationships among the bacterial isolates (Figure 2). Phylogenetic analysis revealed that the strains *Curtobacterium oryzae* AM-38 and *Curtobacterium albidum* AM-42 exhibited 100% homology. The isolate *Tatumella ptyseos* AM-36 showed 99% phylogenetic similarity to strains AM-38 and AM-42. The two *Staphylococcus* species, AM-39 and AM-41, demonstrated 100% homology with each other and exhibited 91% phylogenetic similarity to *Bacillus tropicus* AM-40. Additionally, strains *Calidifontibacillus erzurumensis* AM-46 and *Bacillus aerius* AM-48 showed 92% homology with each other. These findings provide insights into the diversity of endophytic bacteria associated with muscadine cultivars, highlighting both their taxonomic distribution and phylogenetic relationships.

### 3.2. Bacterial Isolates Displayed Variations in Colony Morphology and Cellular Structures

The morphological and microscopic features of nine distinct bacterial isolates were examined, revealing considerable variation in colony appearance, cell shape, and size, as illustrated in Figure 1B,C. On LB agar plates, the colony color ranged from pale to yellow, exhibiting differences among the isolates (Figure 1B). Microscopic examination using DIC imaging and membrane-stained images additionally disclosed differences in cellular morphology. Two strains, AM-39 and AM-41, displayed a cocci shape, whereas the remaining isolates exhibited a rod-shaped structure, albeit with variations in their rod shape, length, and thickness (Figure 1C). Among the isolates, strain AM-40 showed larger colony size as well as wider rod shape. This result indicates a range of morphological diversity across the bacterial strains, suggesting potential differences in their physiological characteristics.

### 3.3. Bacterial Isolates Could Grow at Varying Temperatures

**Figure 3:**
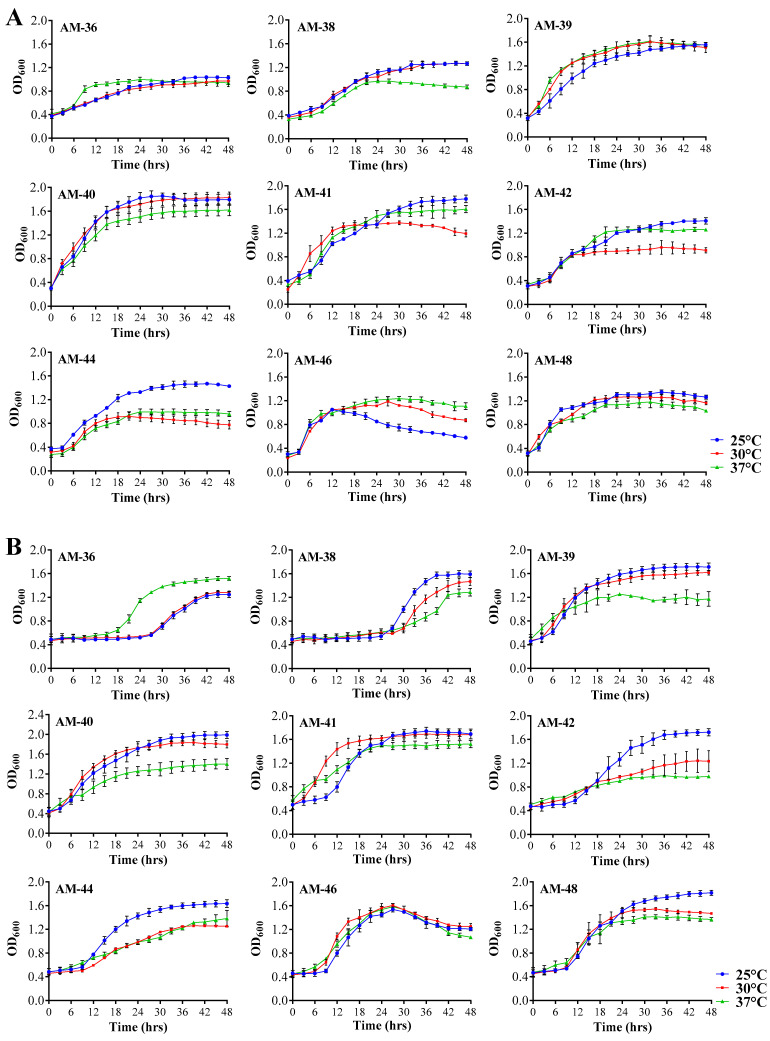
Growth profile of bacterial isolates in different media at varying temperatures. (**A**) Growth profiles of bacterial isolates cultured in LB broth. (**B**) Growth profiles of bacterial isolates cultured in MRS broth. Strains were incubated at three different temperatures: 25 °C, 30 °C, and 37 °C.

Growth curve analysis of the strains in LB and MRS media revealed that all strains were able to survive and grow in both types of media (Figure 3). The growth was monitored at three different temperatures: 25 °C, 30 °C, and 37 °C. Notably, all strains exhibited the ability to sustain growth across all temperatures, with no significant or drastic differences observed between the conditions. Among the nine strains tested, AM-36 and AM-38 demonstrated slower growth patterns and a prolonged lag phase compared to the other strains. This slower growth was consistent in both LB and MRS broth at all temperatures tested. In contrast, AM-40 exhibited the fastest growth across both media types, with notably more rapid growth observed at 25 °C and 30 °C (Figure 3).

### 3.4. Endophytic Isolates Displayed Metabolic Versatility Across Various Biochemical Assays

**Figure 4:**
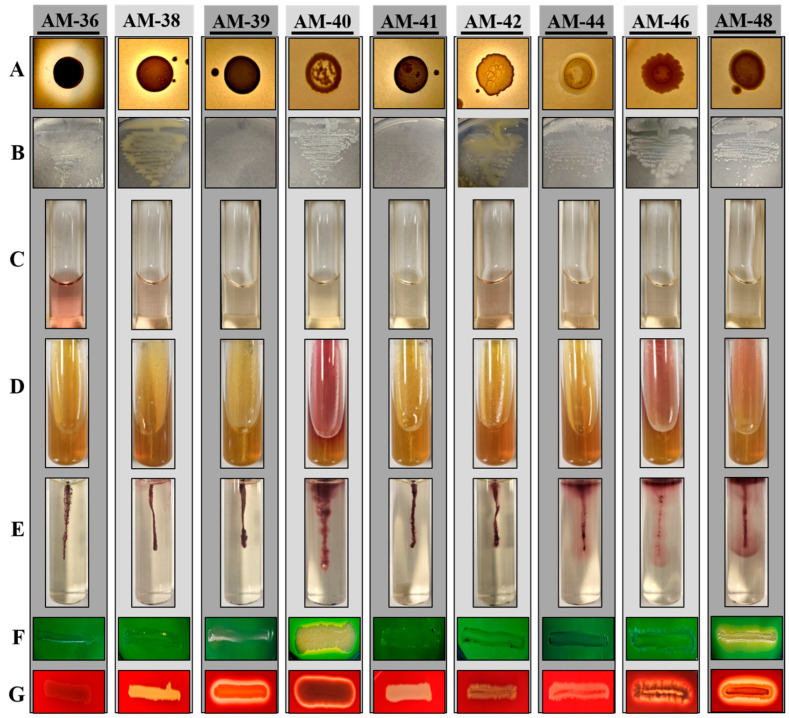
Biochemical characterization of individual bacterial isolates. (**A**) Phosphate solubilization assay, (**B**) screening for nitrogen fixation (**C**) IAA production assay, (**D**) sugar fermentation assay, (**E**) motility assay, (**F**) DNase assay, and (**G**) hemolysis assay. All assays were conducted at least three times, and representative images for each test are shown.

To better understand the functional potential of these isolates, various biochemical tests were conducted to evaluate their capabilities. The ability of the isolates to solubilize phosphate was evaluated using PVK medium. Of the tested isolates, only strains AM-36, AM-38, and AM-42 demonstrated phosphate solubilization, as evidenced by the formation of halo zones around their colonies (Figure 4A, Table S2). Strain AM-36 exhibited rapid solubilization activity, producing a visible halo zone within 2 days of incubation. Similarly, strains AM-38 and AM-42 developed halo zones within 48–56 h. The quantitative measurement of halo zone diameters revealed that strain AM-36 had a significantly larger zone of approximately 0.9 cm, while both AM-38 and AM-42 displayed zones of around 0.5 cm (Figure S1). This finding highlights the superior phosphate solubilization capability of strain AM-36.

The nitrogen fixation ability of the isolates was assessed by culturing them on nitrogen-free media. Most strains exhibited growth within 24 h of incubation, with the exception of AM-39 and AM-41, which showed no detectable growth even after extended incubation periods (Figure 4B, Table S2). Notably, strains AM-36, AM-38, and AM-42 displayed significantly slimy colony morphology on nitrogen-free media compared to their appearance on LB or MRS media, a feature often associated with nitrogen-fixing bacteria.

The assessment of IAA production revealed that strains AM-36, AM-38, and AM-42 exhibited the most pronounced IAA production, as visually evident (Figure 4C). Quantitative spectrophotometric analysis corroborated these observations, ranking the strains in the following order of IAA production potential: AM-36 > AM-38 ≥ AM-42 > AM-44 > AM-46 (Figure S2). These results underscore significant variability in IAA production among the tested isolates, with strain AM-36 displaying the highest capacity. Although strains AM-39, AM-40, AM-48, and AM-41 exhibited some IAA production (Figure S2), they were considered negative due to the very low levels of production (Table S2).

The carbohydrate fermentation capabilities of the isolates were evaluated using TSI media, revealing considerable differences among tested strains. Strains AM-36, AM-39, and AM-41 showed the ability to ferment glucose, lactose, or sucrose. Strains AM-40, AM-46, and AM-48 were positive only for glucose fermentation, while strains AM-38, AM-42, and AM-44 did not exhibit fermentation activity for any of the tested carbohydrates (Figure 4D, Table S2). The oxidase assay revealed that only strains AM-36 and AM-44 were positive for oxidase activity (Table S2), whereas all isolates demonstrated catalase activity (Table S2). These results collectively highlight the metabolic diversity and functional capabilities of the tested isolates, with strain AM-36 emerging as a metabolically versatile and high-performing isolate.

### 3.5. Phylum Bacillota Was Predominant in Motility, DNase, and Hemolytic Assays

The motility assay, designed to assess the ability of bacterial strains to move in a semi-solid medium, revealed distinct variations among the tested isolates. Strains AM-40, AM-44, AM-46, and AM-48 exhibited high motility, indicating their robust movement under the given conditions (Figure 4E). Strain AM-42 also displayed motility, albeit at a significantly lower level. In contrast, strains AM-36, AM-38, AM-39, and AM-41 showed no motility behavior under the same conditions (Figure 4E, Table S2).

To further evaluate the pathogenic potential of these isolates, DNase and hemolysis assays were performed. The DNase assay demonstrated that only strains AM-40 and AM-48 exhibited DNase activity, producing a clear zone around the colonies. All other strains tested negative for DNase activity (Figure 4F, Table S2). The hemolysis assay indicated that strains AM-39, AM-40, AM-46, and AM-48 exhibited significant potency for β-hemolysis. In contrast, the remaining strains did not show any hemolytic activity, and were classified as γ-hemolysis (Figure 4G, Table S2).

### 3.6. Strains AM-40, AM-44, AM-46, and AM-48 Demonstrated Higher Tolerance to Acidic pH

**Figure 5:**
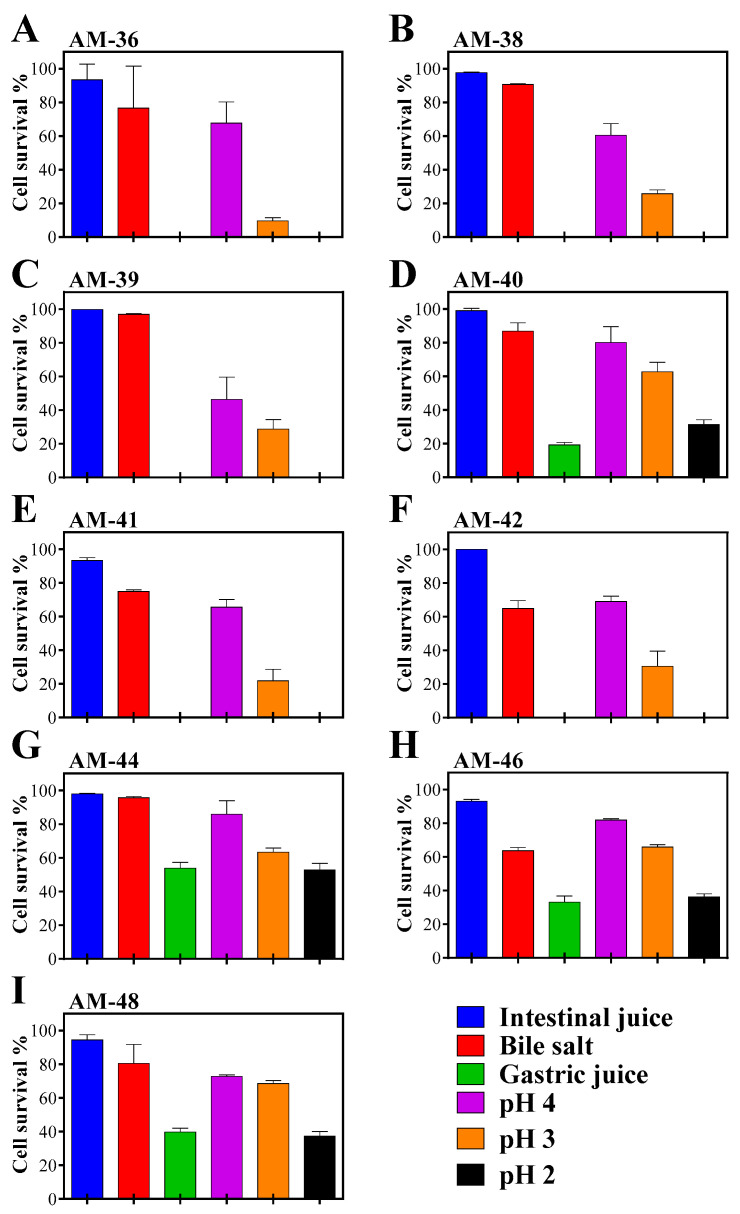
Cell survival assay under gastrointestinal condition. Individual bacterial isolates were incubated in test media for 4 h, and cell viability was determined using the CFU method. (**A**) AM-36, (**B**) AM-38, (**C**) AM-39, (**D**) AM-40, (**E**) AM-41, (**F**) AM-42, (**G**) AM-44, (**H**) AM-46, (**I**) AM-48. The percentage of cell survival was then calculated and shown for each condition.

Since all isolates originated from muscadine grapes, which have a pH of approximately 3.4, we hypothesized their potential to survive under gastrointestinal conditions and serve as probiotics. To evaluate this, we assessed their ability to withstand simulated gut conditions through various tolerance and survival assays. All isolates demonstrated the capacity to survive in intestinal juice after 4 h of incubation, with cell survival rates exceeding 77% (Figure 5A–I, Table S2). Furthermore, all strains exhibited tolerance to 0.3% bile salt, although survival rates varied from 60% to 97%. The highest bile salt survival was observed in strain AM-39, followed by AM-44, AM-38, AM-40, AM-48, AM-36, AM-41, AM-42, and AM-46, as shown in Figure 5. In the presence of gastric juice, only strains AM-40, AM-44, AM-46, and AM-48 were able to survive. Among these, strain AM-44 demonstrated the highest survival rate, followed by AM-48, AM-46, and AM-40 (Figure 5D,G–I).

Acid tolerance testing revealed that all strains survived at pH 4 and pH 3, though with varying survival rates. However, at pH 2, survival rates declined significantly, with only strains AM-40, AM-44, AM-46, and AM-48 demonstrating the ability to tolerate this highly acidic environment. Interestingly, the tolerance pattern at pH 2 mirrored the survival order observed in gastric juice (Figure 5, Table S2).

### 3.7. Isolates Exhibited Distinct Responses for Survival to Varying Stress Conditions

**Figure 6:**
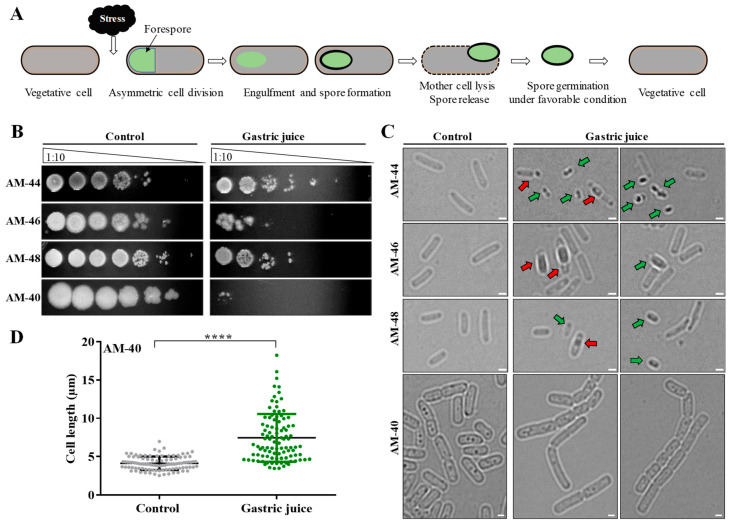
Adaptive responses of bacterial strains for survival under stress conditions. (**A**) Phenomenon of spore formation by *Bacillus* sp. as a stress response. (**B**) Plate assay showing the tolerance of strains to gastric juice after 4 h of incubation along with untreated control. Dilutions were made at a 1:10 ratio. For gastric juice treatment, cells were diluted 100 times before plating the 1st dilution. Note: For strain AM-44, the 5th and 6th dilutions under the gastric juice condition show agar puncture due to the pipette tip, rather than colony growth. (**C**) Representative microscopic images of cells treated with gastric juice compared to untreated control cells. The red arrow indicates a cell bearing a spore, while the green arrow indicates released spores. Scale bar: 1.0 µm. (**D**) Scatter plot showing cell length measurements of strain AM-40, comparing treated and untreated cells. In total, 100 cells were analyzed. *p* values were calculated using one-way ANOVA, with significant differences observed (**** *p* < 0.0001).

The phylum Bacillota is known for its ability to produce spores as a stress response, allowing survival under harsh environmental conditions and subsequent germination in favorable environments (Figure 6A). Given that the strains exhibiting tolerance to low pH conditions belong to this phylum, we investigated whether their ability to form spores contributed to their survival under acidic conditions. To explore this, bacterial cells treated with gastric juice (pH~1.5) were examined microscopically both before and after treatment. Consistent with the results from the cell survival assays (CFU method), the one-plate assay confirmed that strains AM-40, AM-44, AM-46, and AM-48 survived under gastric juice with a similar degree (Figure 6B). Microscopic examinations revealed the presence of spore-bearing cells or released spores in strains AM-44, AM-46, and AM-48, with variation in spore structures among these strains (Figure 6C, Table S2). In contrast, strain AM-40 did not exhibit sporulation under the similar treatment condition. Instead, elongated cells were observed post-treatment, suggesting an alternative survival mechanism (Figure 6C). Further measurement of cell length showed that AM-40 cells nearly doubled in length compared to untreated controls (Figure 6D).

To determine whether strain AM-40 lacked the ability to form spores or exhibited stress-specific responses, all four strains were subjected to nutrient-deprived conditions, and cell structures were examined. The microscopic analysis showed that all strains, including AM-40, produced spores under the tested condition (Figure S3). This finding confirms the ability of strain AM-40 to form spores, while also demonstrating its distinct response to different stress conditions.

## 4. Discussion

Muscadine grapes hold significant promise for various applications, including fresh fruit consumption, wine production, and nutraceutical development, as well as being a resilient crop against pests and diseases. Endophytic microbiota, residing within plant tissues, are hypothesized to play a crucial role in these applications. Despite their importance, the systematic isolation and characterization of bacterial endophytes from muscadine grapes have been poorly addressed. In this study, we have successfully isolated, identified, and characterized bacterial endophytes from muscadine grape berries. Our findings highlight the rich microbial diversity, with six genera identified: *Bacillus*, *Staphylococcus*, *Paenibacillus*, *Calidifontibacillus*, *Curtobacterium*, and *Tatumella*. These genera, except *Calidifontibacillus*, have been reported previously in other grape genotypes (e.g., Bacillus, *Paenibacillus*, and *Staphylococcus* by [44]; Tatumella by [45]; *Curtobacterium* by [46]), emphasizing their widespread presence. Notably, Gram-positive species predominated among the isolates, aligning with previous studies that have reported the abundance of Gram-positive endophytic bacteria [47]. These bacteria are well known for their ability to produce bioactive compounds and exhibit traits such as biocontrol activity, further emphasizing their significance in agricultural and biotechnological applications [47,48]. While bacterial isolate type varied among muscadine cultivars, further studies, including metagenomics and seasonal analyses, are needed to explore their role in phytochemical makeup and genotype-specific variations.

### 4.1. Functional Diversity and Biotechnological Potential of Isolates

**Figure 7:**
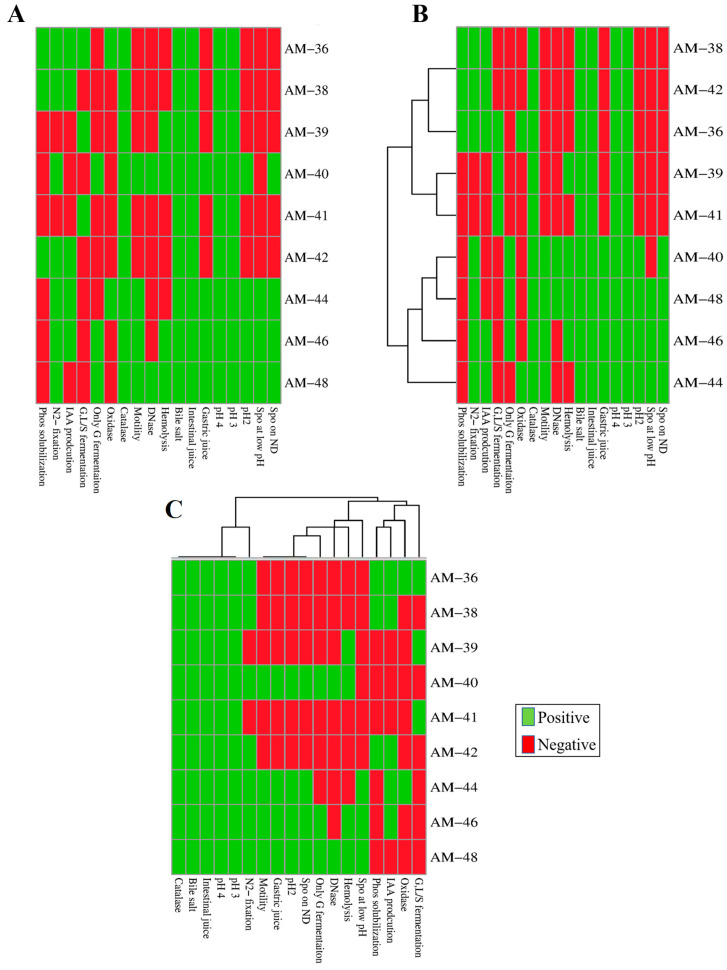
Biochemical test results and cluster analysis of bacterial isolates. (**A**) Heatmap depicting the positive (green) and negative (red) outcomes of biochemical tests across bacterial isolates and functional traits. The x-axis lists individual isolates, while the y-axis corresponds to functional traits. (**B**) Hierarchical clustering dendrogram of bacterial isolates based on their biochemical test profiles, grouping isolates with similar functional traits. (**C**) Hierarchical clustering dendrogram of functional traits, grouping traits with similar patterns across isolates. Abbreviations: Spo—Sporulation; ND—Nutrient Depletion; Phos—Phosphate; G—Glucose; G, L/S—Glucose, Lactose, or Sucrose, N_2_—Nitrogen.

Biochemical characterization studies conducted on these isolates revealed a broad spectrum of results (Figure 7A). For instance, strain *Tatumella ptyseos* AM-36 demonstrated significant abilities in phosphate solubilization, N_2_-fixation, and IAA production, along with positive oxidase and catalase activity, making it a suitable candidate for promoting plant growth and mitigating abiotic stress. Additionally, the strain exhibited the ability to ferment glucose, lactose, or sucrose. Previous studies have reported the abundance of this species during the spontaneous fermentation of wine, underscoring its potential applications in vinification, ethanol production, and enhancing wine sensory attributes [49,50]. Importantly, it was deemed safe for application, as it tested negative for DNase and hemolytic activity.

Two *Curtobacterium* spp., AM-38 and AM-42, which share 100% homology in their phylogenetic relation, exhibited similar biochemical profiles, underscoring their potential as candidates for plant growth promotion. Previous studies have also highlighted plant growth-promoting traits within the *Curtobacterium* genus [51]. Moreover, *Curtobacterium* has shown a strong ability to suppress plant diseases, such as crown gall development [52], further suggesting its potential as a biocontrol agent in agricultural applications.

Strains AM-39 and AM-41, belonging to the genus *Staphylococcus*, displayed carbohydrate fermentation abilities but lacked significant plant growth-promoting traits. Although both strains shared 100% homology and similar biochemical profiles, a notable distinction was observed in their hemolytic activities. Strain AM-39 tested positive for hemolysis, while AM-41 was non-hemolytic. The *Staphylococcus* genus has also been widely reported as a prominent microbial group in grapevines and integrated pest management vineyards [47,53]. Interestingly, certain species of coagulase-negative *Staphylococcus* have been proposed as starter cultures for various fermented foods [54]. Furthermore, *Staphylococcus* species have been associated with the production of staphylococcins, a class of lantibiotics with various applications, including bactericidal use, clinical treatments, food biopreservation, and agriculture [55].

*Bacillus* spp. AM-40 and AM-48 exhibited distinct characteristics, with AM-40 forming larger colonies and displaying faster growth with a shorter lag phase. Both strains tested positive for N_2_-fixation and glucose fermentation, indicating their potential to contribute to plant nutrient acquisition. Additionally, both strains showed motility behavior along with their DNase and hemolytic activities. *Bacillus* species are well documented for their role in promoting plant growth and suppressing plant pathogens, including grape gray mold, ripe rot, grape rot, and downy mildew [22,56,57,58,59,60,61]. Notably, the rapid growth and motility observed in these strains are key traits of the biocontrol mechanisms employed by endophytic bacteria, which enable effective colonization and competition for nutrients and space, thereby reducing the incidence of plant diseases [62].

Isolate AM-44, belonging to genus *Paenibacillus*, a genus related to *Bacillus*, demonstrated N_2_-fixation ability and low-level IAA production, though it lacked carbohydrate fermentation ability. This strain exhibited motility but tested negative for DNase and hemolysis, confirming its safety for application. Previous studies have highlighted the potential of *Paenibacillus* species as effective plant growth-promoting bacteria and biocontrol agents against plant diseases [63,64,65]. Moreover, *Paenibacillus* species isolated from grapevines have been shown to produce specific volatile aromatic compounds which influence the aroma and flavor of wine [66,67], suggesting their role in oenological applications as well.

Strain *C. erzurumensis* AM-46, belonging to the novel genus *Calidifontibacillus* within the family Bacillaceae, showed close phylogenetic homology with *Bacillus aerius*. It shared similar traits with *B. aerius* but was DNase-negative, indicating a potential difference in safety or functionality. One of the most remarkable traits of AM-46 was its high motility, the highest observed among all tested strains. Motility also facilitates the formation of biofilms, a feature that enhances plant–microbe interactions and supports plant health [68]. This genus has not been previously reported as endophytic, making the discovery of *C. erzurumensis* AM-46 in this study noteworthy. Its association with plants and demonstrated traits hint at its potential to function as a biocontrol agent, similar to other *Bacillus* species.

### 4.2. Adaptive Survival Strategies of Potential Probiotic Isolates Under Simulated Gastrointestinal Stress Conditions

The ability of probiotics to survive and function in the gastrointestinal condition is a significant factor determining their effectiveness. In this study, all isolates demonstrated the ability to survive at 37 °C, in intestinal juice and bile salts, indicating their adaptability to environments mimicking intestinal conditions. Interestingly, the survival of these isolates at pH 3 and 4 aligns with the natural acidic environment of grape berries, suggesting an intrinsic tolerance to moderately acidic conditions. Isolates belonging to the families Bacillaceae and Paenibacillaceae demonstrated exceptional resilience, surviving pH levels below 3, and gastric juice. This suggests that these strains possess intrinsic mechanisms for coping with acidic stress, making them promising candidates for probiotic applications. It is important to note that while all four strains demonstrated the ability to survive under simulated gastric conditions, only strain *Paenibacillus cineris* AM-44 was deemed safe and nonpathogenic for probiotic applications, as evidenced by its negative results in both DNase and hemolysis tests. Supporting this finding, a previous study showed that related species of *Paenibacillus* possess probiotic characteristics and beneficial effects on intestinal microbiota in poultry [69].

Furthermore, the diverse survival strategies exhibited by these isolates, including filamentation/cell elongation and sporulation, underscore the adaptability of microbes to different environmental challenges. For example, *Paraburkholderia elongata* exhibits filamentation in response to elevated phosphate levels, playing a crucial role in nutrient cycling and soil biogeochemistry [70]. Similarly, *Stenotrophomonas* sp. demonstrates filamentation as a survival mechanism under metal stress, further underscoring the ecological importance of this adaptation [43]. Conversely, sporulation is a highly regulated developmental process that enables the formation of resistant endospores. The resilience of spores has significant practical applications, particularly in probiotics and agricultural biocontrol agents, where they contribute to the long-term viability of beneficial bacterial strains [71,72].

**Figure 8:**
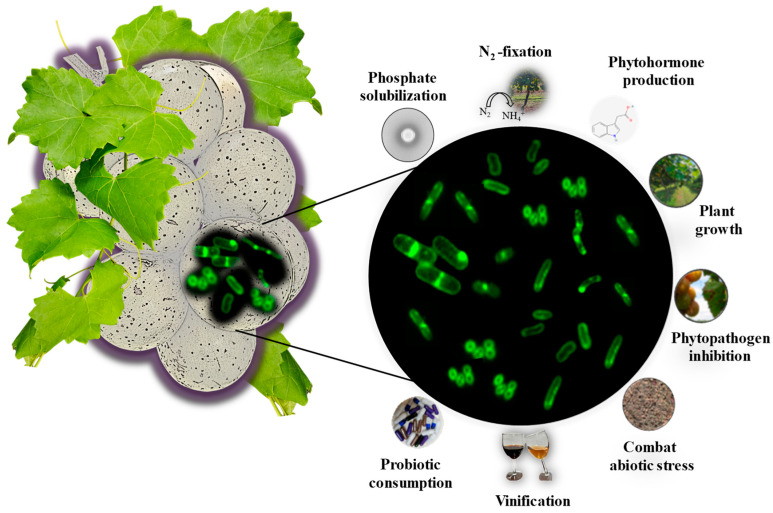
Potential function of muscadine grape berry bacterial endophytes. Based on the traits exhibited by the bacterial isolates, their potential functions for various applications were predicted. These functions include roles in nutrient cycling, stress tolerance, plant growth promotion, vinification, and biotechnological applications, highlighting their versatility in different environmental and industrial contexts.

Altogether, the biochemical assays highlighted diverse traits exhibited by these isolates. A cluster-based analysis of the bacterial strains revealed distinct grouping patterns: strains AM-38, AM-42, and AM-36 grouped closely together, while AM-39 and AM-41 formed a nearby branching cluster. In contrast, strains AM-40, AM-48, AM-46, and AM-44 clustered separately, suggesting potential functional specialization (Figure 7B). Further cluster analysis based on physiological traits showed that all strains exhibited consistent catalase activity and demonstrated tolerance to intestinal juice, bile salts, and pH 4 and pH 3, though with varying degrees of efficiency. These findings indicate a robust adaptive capacity among the strains, while the observed variations in other assays highlight their functional diversity (Figure 7C). Overall, the metabolic functions and adaptive strategies of the isolates highlight the versatility of bacteria in responding to environmental pressures, emphasizing their significant potential for supporting host plants and advancing biotechnological applications (Figure 8). Understanding these mechanisms provides a foundation for leveraging bacterial traits in innovative industrial processes and sustainable environmental solutions.

## 5. Conclusions

This research significantly enhances our understanding of bacterial endophytes in muscadine grapes and paves the way for their potential applications in agriculture, biotechnology, and probiotic development. Our study is the first to explore the cultivable endophytic bacterial diversity across different muscadine grape cultivars, offering valuable insights into their isolation, molecular identification, taxonomic distribution, phylogenetic relationships, and morphological, physiological, and biochemical characteristics. Additionally, the establishment of a strain stock repository and pathogenicity assessment further contribute to the systematic study of these beneficial microbes. Key findings include the identification of *Tatumella ptyseos* as a highly effective plant growth promoter and the recently discovered *Calidifontibacillus erzurumensis* as a novel endophytic species with potential roles in mitigating biotic stress. Additionally, *Bacillus* spp. and *Paenibacillus cineris* emerged as potential candidates for biocontrol and probiotic applications. While this study provides a comprehensive overview of the functional roles of these bacterial endophytes, further research is required to investigate their detailed mechanistic functions and quantify their contributions to plant health and disease resistance. Expanding on these findings will be crucial for fully understanding the potential of these microbes in agricultural practices and their broader biotechnological applications.

## Figures and Tables

**Table 1:** Characteristics of muscadine grape cultivars and bacterial species isolated. The table summarizes the muscadine grape cultivars used in this study, including their berry color, pH, and Brix levels. Additionally, it lists the bacterial species isolated from the berries along with their Gram stain characteristics.

Muscadine Grape Cultivar	Berry Color	Berry pH	Berry Brix	Bacterial Isolate Identified	Gram Stain
Late fry	Bronze	3.22 ± 0.09	16.5 ± 0.4	*Tatumella ptyseos* AM-36	Negative
Summit	Bronze	3.47 ± 0.16	17.13 ± 0.32	*Tatumella ptyseos* AM-37	Negative
Black fry	Black	3.34 ± 0.12	16.33 ± 0.32	*Curtobacterium oryzae* AM-38	Positive
Cowart	Black	3.61 ± 0.18	15.56 ± 0.21	*Staphylococcus aureus* AM-39	Positive
				*Bacillus tropicus* AM-40	Positive
Jumbo	Black	3.42 ± 0.09	17.6 ± 0.36	*Staphylococcus warneri* AM-41	Positive
				*Curtobacterium citreum* AM-42	Positive
Scuppernong	Bronze	3.52 ± 0.12	15.26 ± 0.15	*Curtobacterium oryzae* AM-43	Positive
Welder	Bronze	3.31 ± 0.07	17.53 ± 0.40	*Paenibacillus cineris* AM-44	Positive
Tara	Bronze	3.53 ± 0.05	15.36 ± 0.41	*Calidifontibacillus erzurumensis* AM-45	Positive
Hunt	Black	3.35 ± 0.04	15.5 ± 0.36	*Calidifontibacillus erzurumensis* AM-46	Positive
Doreen	Bronze	3.29 ± 0.04	13.46 ± 0.25	*Staphylococcus warneri* AM-47	Positive
				*Bacillus aerius* AM-48	Positive
Southern Home	Black	3.67 ± 0.07	16.5 ± 0.4	*Calidifontibacillus erzurumensis* AM-49	Positive
Southland	Black	3.68 ± 0.09	15.56 ± 0.21	*Staphylococcus warneri* AM-50	Positive

**Table 2:** GenBank accession number of bacterial isolates characterized in this study.

Bacterial Isolate	Accession Number
*Tatumella ptyseos* AM-36	PP915744
*Curtobacterium oryzae* AM-38	PP916015
*Staphylococcus aureus* AM-39	PP916060
*Bacillus tropicus* AM-40	PP916016
*Staphylococcus warneri* AM-41	PP916017
*Curtobacterium citreum* AM-42	PP916043
*Paenibacillus cineris* AM-44	PP916050
*Calidifontibacillus erzurumensis* AM-46	PP916051
*Bacillus aerius* AM-48	PP916056

## Data Availability

The data supporting the findings of this study are publicly available in NCBI database, with the corresponding accession numbers provided within the article.

## Supplementary Materials

The following supporting information can be downloaded at: https://www.mdpi.com/article/10.3390/cells14050369/s1, Figure S1: Phosphate solubilization by strains AM-36, AM-38, and AM-42. (A) Measurement of halo zones produced by strains AM-36, AM-38, and AM-42 on PVK plates. Images were captured after 7 days of incubation. (B) Graph representing the colony diameter of the phosphate solubilizing zone displayed by each bacterial strain. The experiment was done in triplicate and error bars represented as standard deviation. Figure S2: Higher IAA production by strain AM-36. (A) Representative images of test tubes showing the color generated in the IAA production assay for individual strains, along with the control tube. (B) Graph representing the spectrometric readings of the color intensity generated by each bacterial strain. The experiment was done in triplicate and error bars represented as standard deviation. Figure S3: Strains belonging to *Bacillaceae* and *Paenibacillaceae* families exhibited spore formation under nutrient depletion condition. (A) AM-40, (B) AM-44, (C) AM-46, (D) AM-48. Strains were incubated in nutrient-depleting conditions, followed by microscopic examination. The green arrow indicates the presence of spore structures. Scale bar: 1.0 µm. Table S1: Classification of bacterial isolates at the genus, family, and phylum levels. Table S2: Result of biochemical tests conducted on the bacterial isolates in this study. This table displays the outcomes of various biochemical tests performed on the bacterial isolates, indicating their positive or negative responses to each assay.
