# Supplementary material for: Isolation and Functional Characterization of Endophytic Bacteria from Muscadine Grape Berries: A Microbial Treasure Trove
Source: Cells. 2025 Mar 3;14(5):369. doi: 10.3390/cells14050369 (PMC11899604; doi:10.3390/cells14050369)
Supplement: Supplementary file 1 [file cells-14-00369-s001.zip › cells-3499090-supplementary.pdf]

Supplementary information:

Table S1. Classification of bacterial isolates at the genus, family, and phylum levels.

Strain	Genus	Family	Phylum
Tatumella ptyseos AM-36	*Tatumella*	*Erwiniaceae*	Pseudomonadota
Curtobacterium oryzae AM-38	*Curtobacterium*	*Microbacteriaceae*	Actinomycetota
Staphylococcus aureus AM-39	*Staphylococcus*	*Staphylococcaceae*	Bacillota
Bacillus tropicus AM-40	*Bacillus*	*Bacillaceae*	Bacillota
Staphylococcus warneri AM-41	*Staphylococcus*	*Staphylococcaceae*	Bacillota
Curtobacterium citreum AM-42	*Curtobacterium*	*Microbacteriaceae*	Actinomycetota
Paenibacillus cineris AM-44	*Paenibacillus*	*Paenibacillaceae*	Bacillota
Calidifontibacillus erzurumensis AM-46	*Calidifontibacillus*	*Bacillaceae*	Bacillota
Bacillus aerius AM-48	*Bacillus*	*Bacillaceae*	Bacillota

Table S2. Result of biochemical tests conducted on the bacterial isolates in this study. This table displays the outcomes of various biochemical tests performed on the bacterial isolates, indicating their positive or negative responses to each assay.

**Biochemical Tests**	**AM -36**	**AM -38**	**AM-39**	**AM-40**	**AM-41**	**AM-42**	**AM-44**	**AM-46**	**AM-48**
Phosphate solubilization	+	+	-	-	-	+	-	-	-
Nitrogen fixation	+	+	-	+	-	+	+	+	+
IAA production	+	+	-	-	-	+	+	+	-
Carbohydrate fermentation	G, L/S	-	G, L/S	G	G, L/S	-	-	G	G
Oxidase	+	-	-	-	-	-	+	-	-
Catalase	+	+	+	+	+	+	+	+	+
Motility	-	-	-	+	-	-	+	+	+
DNase	-	-	-	+	-	-	-	-	+
Hemolysis	γ	γ	β	β	γ	γ	γ	β	β
Bile salt	+	+	+	+	+	+	+	+	+
Intestinal juice	+	+	+	+	+	+	+	+	+
Gastric juice	-	-	-	+	-	-	+	+	+
pH 4	+	+	+	+	+	+	+	+	+
pH 3	+	+	+	+	+	+	+	+	+
pH 2	-	-	-	+	-	-	+	+	+
Sporulation at low pH	-	-	-	-	-	-	+	+	+
Sporulation on nutrient depletion	-	-	-	+	-	-	+	+	+

γ - Gamma hemolysis, β- No hemolysis, L- Lactose fermentation, S- Sucrose fermentation, G- Glucose fermentation, + Positive, - Negative,

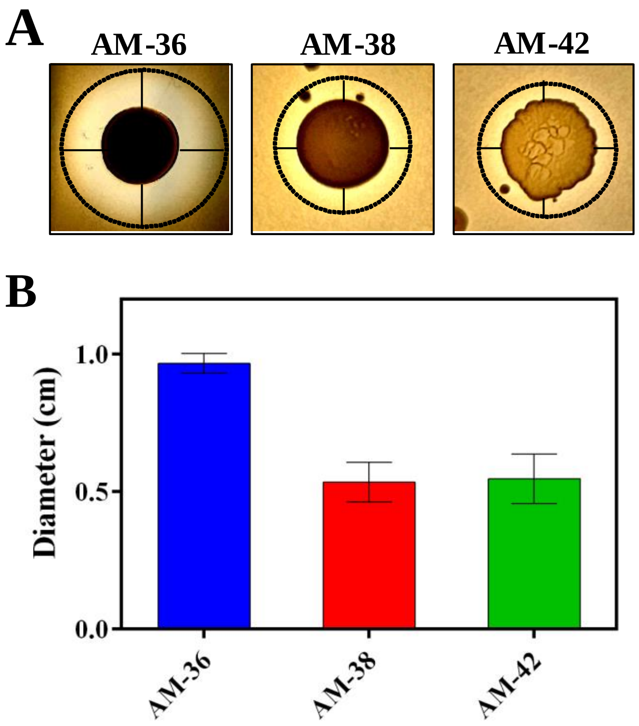

Figure S1. Phosphate solubilization by strains AM-36, AM-38, and AM-42. (**A**) Measurement of halo zones produced by strains AM-36, AM-38, and AM-42 on PVK plates. Images were captured after 7 days of incubation. (**B**) Graph representing the colony diameter of the phosphate solubilizing zone displayed by each bacterial strain. The experiment was done in triplicate and error bars represented as standard deviation.

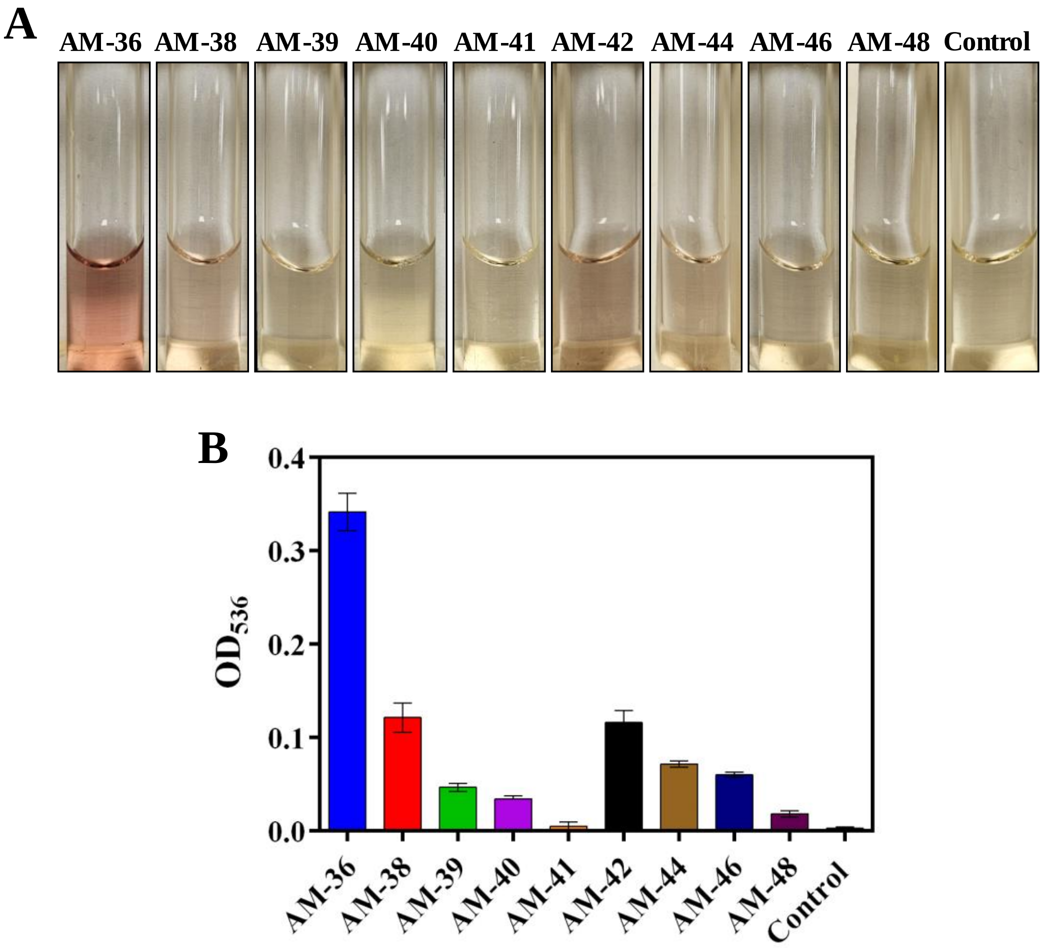

Figure S2. Higher IAA production by strain AM-36. (**A**) Representative images of test tubes showing the color generated in the IAA production assay for individual strains, along with the control tube. (**B**) Graph representing the spectrometric readings of the color intensity generated by each bacterial strain. The experiment was done in triplicate and error bars represented as standard deviation.

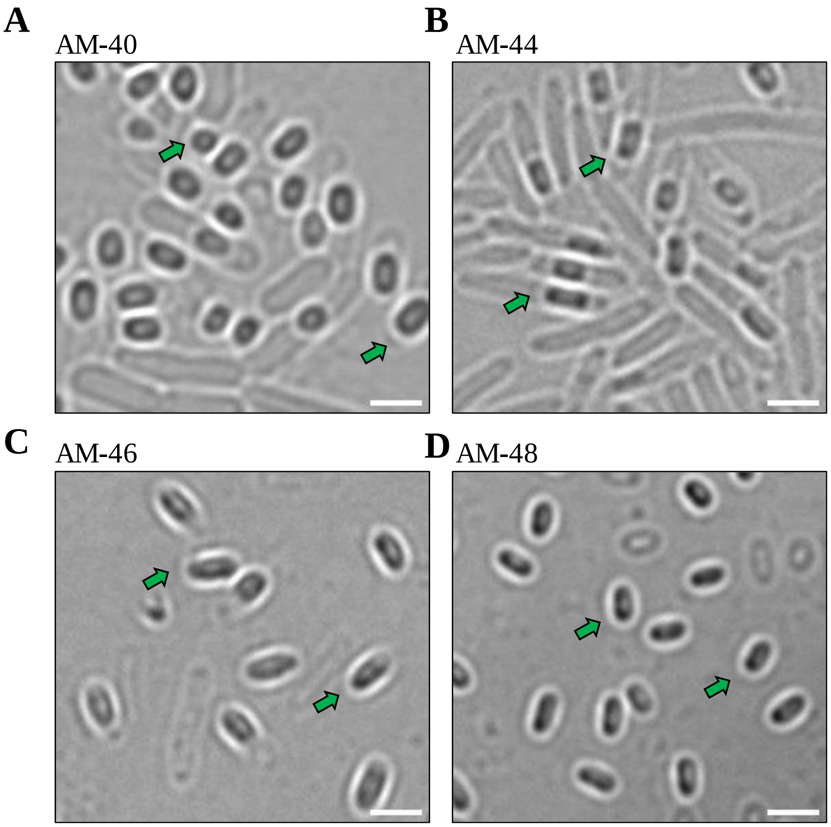

Figure S3. Strains belonging to *Bacillaceae* and *Paenibacillaceae* families exhibited spore formation under nutrient depletion condition. (**A**) AM-40, (**B**) AM-44, (**C**) AM-46, (**D**) AM-48. Strains were incubated in nutrient-depleting conditions, followed by microscopic examination. The green arrow indicates the presence of spore structures. Scale bar: 1.0 µm.
